# Alzheimer’s disease and epilepsy: shared neuropathology guides current and future treatment strategies

**DOI:** 10.3389/fneur.2023.1241339

**Published:** 2023-10-23

**Authors:** Olivia Lu, Taimur Kouser, Irina A. Skylar-Scott

**Affiliations:** ^1^Stanford Neuroscience Clinical Research Group, Department of Neurology and Neurological Sciences, Stanford University School of Medicine, Palo Alto, CA, United States; ^2^Stanford University School of Medicine, Palo Alto, CA, United States; ^3^Memory Disorders Division, Department of Neurology and Neurological Sciences, Stanford University School of Medicine, Palo Alto, CA, United States

**Keywords:** Alzheimer’s disease, epilepsy, seizures, cortical irritability, epileptiform discharges, management, treatment, therapeutic pipeline

## Abstract

Epilepsy is a cause of profound disability in patients with Alzheimer’s disease (AD). The risk of being diagnosed with AD increases the risk for epilepsy, and in parallel, a history of epilepsy increases the likelihood of the development of AD. This bi-directional relationship may be due to underlying shared pathophysiologic hallmarks, including decreased cerebrospinal fluid amyloid beta 42 (Aβ42), increased hyperphosphorylated tau protein, and hippocampal hyperexcitability. Additionally, there are practical treatment considerations in patients with co-morbid AD and epilepsy—namely, there is a higher risk of seizures associated with medications commonly prescribed for Alzheimer’s disease patients, including antidepressants and antipsychotics such as trazodone, serotonin norepinephrine reuptake inhibitors (SNRIs), and first-generation neuroleptics. Anti-amyloid antibodies like aducanumab and lecanemab present new and unique considerations in patients with co-morbid AD and epilepsy given the risk of seizures associated with amyloid-related imaging abnormalities (ARIA) seen with this drug class. Finally, we identify and detail five active studies, including two clinical trials of levetiracetam in the respective treatment of cognition and neuropsychiatric features of AD, a study characterizing the prevalence of epilepsy in AD via prolonged EEG monitoring, a study characterizing AD biomarkers in late-onset epilepsy, and a study evaluating hyperexcitability in AD. These ongoing trials may guide future clinical decision-making and the development of novel therapeutics.

## Introduction

Alzheimer’s disease (AD) and epilepsy exact a profound mental, emotional, and physical toll on patients and caregivers. AD and epilepsy affect 24 million and 50 million people worldwide, respectively (1). As many as 10–22% of patients living with AD will have at least one seizure, and about two-thirds of those patients will have recurrent seizures without a clear acute cause 24 or more hours apart, or epilepsy (2–4). The International League Against Epilepsy (ILAE) includes two other presentations under the umbrella of epilepsy: an unprovoked seizure and a probability of ≥60% of a recurrent seizure or an epilepsy syndrome. The ILAE also has an operational definition of an epileptic seizure, used in this review, which is a transient manifestation of signs and/or symptoms due to abnormal excessive or synchronous activity of neurons in the brain (5). The National Institute of Aging-Alzheimer’s Association workgroups have developed consensus criteria for probable AD dementia with an amnestic presentation, and it is diagnosed when patients: (1) meet criteria for dementia; (2) exhibit insidious onset; (3) have a clear-cut history of declining cognition; and (4) the initial and most prominent cognitive deficit is impairment in learning and recall of information that has been recently learned as well as cognitive dysfunction in one other cognitive domain, and this criteria is used herein. There are also non-amnestic presentations of atypical AD where other cognitive domains are impaired early and prominently (6).

The association between epilepsy and AD appears to be bi-directional, wherein a diagnosis of epilepsy is associated with approximately 2-fold greater odds of both subsequent all-cause dementia and AD based on a systematic review of 20 longitudinal studies (7). Additionally, in the same systematic review, both all-cause dementia and AD were associated with 3-fold greater odds of developing epilepsy (7). Vascular risk factors may modulate this effect; they may slightly decrease the risk of dementia in those with pre-existing seizures and, conversely, increase the risk of the development of seizures in those with a diagnosis of dementia (8). These neurologic conditions have shared pathophysiological features which may underlie this bi-directional association. Additionally, when they occur in concert, there are unique clinical and treatment considerations in these patients that providers need to consider and researchers continue to explore (Table 1).

**Table 1:** Summary of high-quality studies related to therapeutic considerations in patients with Alzheimer’s disease and co-morbid seizures or epileptiform activity.

Study	Study type	*N*	Age	*N* (%) epileptiform activity	*N* (%) seizures	Risks and outcomes
Vossel et al. (9)	Phase 2a crossover RCT for levetiracetam	34	62.3 ± 7.7	Crossover trial: Placebo-levetiracetam: 4 (26.7) Levetiracetam-placebo: 7 (41.2)	0	Levetiracetam treatment did not result in improvement in its primary outcome measure (test of executive function). Improved accuracy in exploratory analyses (spatial memory and executive functioning tasks) among participants with epileptiform activity
Meador et al. (10)	An RCT assessing the neurocognitive effects of brivaracetam, levetiracetam, and lorazepam in healthy volunteers	16	18–50	N/A	N/A	Lorazepam adversely affected the CNT score, a composite of EEG, evoked potentials and cognitive tests
Taipale et al. (11)	Case-control study to evaluate the association between regular ASM use and incident dementia	20,325 (dementia of any type) 70,718 (AD)	75.7 ± 6.7 (dementia of any type) 78.1 ± 7.1 (AD)	N/A	N/A	Regular use of phenobarbital, carbamazepine, phenytoin, valproate, primidone, barbexaclone, ethosuximide, clonazepam, zonisamide, and topiramate was associated with a greater risk of incident dementia and AD, 28 and 15%, respectively compared with controls not on AEDs
Cumbo and Ligori (12)	A prospective, randomized, three-arm parallel-group, case–control study for levetiracetam, phenobarbital, and lamotrigine	95	60–90	N/A	(Average N per month) LEV: 5.52 PB: 5.71 LTG 5.65	Levetiracetam group exhibited increased MMSE scores and lamotrigine improved mood (Cornell scale for depression)
Hill et al. (13)	Cohort study to assess associations between antidepressant and seizures	238,963	20–64	N/A	3,325 (1.39)	HR for seizures significantly increased for all antidepressant classes (highest risk: trazodone, lofepramine, and venlafaxine)
Chu et al. (14)	A case-control study to investigate the association between exposure to antidepressants and risk of epilepsy	863	48.12 (18.56) (with epilepsy)	N/A	N/A	Patients with depression using SSRIs or SNRIs were two times more likely to develop first-time seizures compared with non-users. Risk of epilepsy increases with longer antidepressant treatment duration
Bloechliger et al. (15)	A nested case-control analysis of the association between antipsychotic drug use and the development of first-time seizures in patients with schizophrenia, affective disorders, or dementia	60,121	Unknown	N/A	N/A	Patients with dementia had significantly higher incidence rates of first-time seizures, compared with patients with other affective disorders. Drugs such as olanzapine or quetiapine increased risk of seizures
Hamberger et al. (16)	An RCT of donepezil to improve memory in epilepsy	23 (with subjective memory concerns)	18–55	N/A	(Average N per month for donepezil) 2.6 (5.7)	Donepezil treatment did not result in improvement in seizure frequency or severity. Treatment also had no significant effect on cognitive scores
Ha et al. (17)	A population-based study for incident seizure in dementia (AD or vascular dementia)	13,767	65–95	N/A	N/A	A slight increase in seizure risk for patients receiving donepezil for 1 year compared to memantine

RCT, randomized controlled trial; CNT, cognitive neuropsychological test; ASM, antiseizure medication; MMSE, Mini-Mental State Examination; HR, hazard ratio; AD, Alzheimer’s disease.

## Methods

The authors completed a literature review of publications describing the relevant epidemiology, pathophysiology, semiology, and treatment considerations for patients with AD and epilepsy. Research studies were carefully reviewed and selected on the basis of their relevance to this topic (including seizures and Alzheimer’s disease, epilepsy and Alzheimer’s disease, epileptiform discharges and AD, epileptiform activity and AD, these syndromes and epidemiology, these syndromes and pathophysiology, and these syndromes and treatment). The search was completed using PubMed and Google Scholar with a particular emphasis on articles from the last 5 years and inclusion of older, important studies as well. A search of clinicaltrials.gov for clinical trials in the pipeline was also completed.

## Shared pathology of Alzheimer’s disease and epilepsy

### Amyloid

Emerging evidence indicates that AD and epilepsy have shared neuropathological hallmarks. In one study, for example, 40 individuals with a mean age of 70 with late-onset epilepsy of unknown origin (LOEU), which is defined as epilepsy onset over the age of 65 without a clear secondary cause, had significantly lower levels of cerebrospinal fluid amyloid beta 42 (CSF Aβ42) compared to healthy age-matched controls (703.9 ± 388.3 pg./mL in LOEU vs. 975 ± 275 pg./mL in controls, expressed as mean ± standard deviation) (18, 19). Alzheimer’s disease is similarly characterized by low levels of Aβ42 in the CSF, which is thought to reflect Aβ42 aggregation in amyloid plaques in the brain (20). In this study, Aβ42 levels lower than 500 pg./mL was considered pathologic (i.e., in the Alzheimer’s range), so both those with epilepsy and those without remained, on average, in the normal range (19).

### Tau

In a clinicopathological study, researchers found that hyperphosphorylated tau pathology, as identified by immunohistochemical stains of tissue from temporal lobe resections, was associated with cognitive decline in 31 of 33 individuals with temporal lobe epilepsy (TLE). Hyperphosphorylated tau is the pathological isoform of tau protein that accumulates in the form of neurofibrillary tangles (NFTs) in AD. The participants, all of whom were between ages 50 and 65, underwent temporal lobe resection for refractory TLE. A heavier burden of tau pathology in tissue was associated with a greater decline in verbal learning, verbal recall, and naming, when comparing pre- and post-temporal lobe resection cognitive evaluations 1 year apart. Additionally, higher tau levels in tissue were also associated with a greater likelihood of secondary generalization prior to resection (21, 22). The fact that these individuals had lifelong epilepsy and temporal lobe resections may mean that these results do not generalize to most patients with AD who develop seizures, but the identification of phosphorylated tau in the brain tissue of patients with chronic epilepsy does underscore the potential shared neuropathology between patients with chronic epilepsy and those with AD. Indeed, several studies now suggest that epilepsy in AD and TLE share common neuropathological pathways (23).

In the largest database study to date of biochemical markers of AD and epilepsy, 17,901 patients with CSF tau and a diagnosis of Alzheimer’s disease were identified; of these, 851 were also diagnosed with epilepsy. Patients in this study with both epilepsy and AD had higher levels of both total tau and phosphorylated tau in CSF compared with patients with AD who did not have co-morbid epilepsy. Additionally, CSF Aβ42 levels were lower in patients with both diagnoses compared with those with AD alone. These findings strongly suggest that a higher burden of AD pathology is associated with a higher risk of epilepsy and Alzheimer’s disease dual diagnoses (24).

In a cohort study of 292 patients with AD and CSF testing who were followed for a mean of 5 years, almost 18% had a first-time seizure. In a univariate analysis, the development of seizures was associated with CSF total tau levels but not with CSF hyperphosphorylated tau or amyloid-β, unlike the database study detailed above. This may suggest greater cortical structural damage in patients with both AD and seizures compared with patients with AD who remain seizure-free. In a Cox regression, the probability of a seizure was associated with CSF total tau but not CSF hyperphosphorylated tau or amyloid-β, which may suggest tau-induced cortical irritability. Of note, this study was completed in a relatively young cohort for typical AD; the mean age of AD onset was 59 in the patients with seizures and almost 65 in those without seizures (25).

### Brain atrophy

In terms of morphology, a study of 73 participants over the age of 55 with TLE demonstrated a analogous pattern and degree of atrophy of the medial temporal lobes compared to individuals with amnestic mild cognitive impairment (aMCI), an at risk stage for AD dementia (23). Both TLE and aMCI groups showed significant impairment in memory encoding, naming, and category fluency relative to healthy controls (26). These morphologic changes in patients with TLE may lower cognitive reserve and partially explain the increased predisposition of patients with epilepsy to get AD.

Thus, epilepsy, particularly in late life, and AD may share common underlying neuropathology which can serve as a target for therapeutic approaches for both diseases, as detailed in the sections on management below.

## Epileptiform discharges and high frequency oscillations in Alzheimer’s disease

### Epileptiform discharges

Subclinical epileptiform activity (SEA) refers to the presence of epileptiform discharges, specifically spikes or sharp waves, in patients without known seizures (27). There is substantial variability in the published prevalence rates of patients living with AD who have SEA (between 3 and 54%) (28). In one study, SEA was assessed in 19 cognitively normal controls and 33 age-matched participants with a mean age of 62 years who met criteria for AD and did not have a history of seizures. SEA was detected in 42% of participants with AD, which was more than four times the detection rate in controls. Of note, 90% of epileptiform discharges in individuals with AD were detected during EEG recordings of sleep. Over an average of 3.3 years, participants with SEA declined significantly on the Mini-Mental State Examination (MMSE) by 3.9 points per year (vs. 1.6 points/year in patients with AD who did not have SEA) and on an executive function composite (29).

A growing body of literature has also evaluated interictal epileptiform activity (IEA), which refers to epileptiform discharges between seizures, and its prevalence in AD. In one study evaluating IEA prevalence, 10 of 48 patients with AD evaluated with 24-h EEG were diagnosed with seizures, and 80% of those patients had IEA (30). In a small case series of two patients with AD who wore foramen ovale electrodes near the temporal lobes, the electrodes detected epileptiform discharges and silent hippocampal seizures during sleep. These patients did not have a clinical history of seizures, and sleep is considered a critical period for memory consolidation. The authors suggest that hippocampal hyperexcitability may contribute to the pathophysiology of AD, but this remains to be proven (31).

It remains unclear whether addressing IEA or SEA may help in the treatment or prevention of cognitive decline in AD, but like the authors of the case series above, some advocate for considering IEA and SEA part of the pathophysiological influences that drive cognitive impairment in AD. Hypothesized mechanisms include a compromised glutamatergic system, excitotoxicity-induced neurodegeneration, accelerated amyloid and tau deposition driven by epileptiform discharges, remodeling due to hyperexcitability resulting in disconnection of functional networks, and alteration of sleep structure, among others (32). Because patients with AD who have SEA experience faster worsening of executive function and global measures of cognition than those without, one phase 2a trial looked at the effect of levetiracetam on 34 participants with probable AD. The study, known as the Levetiracetam for Alzheimer’s Disease–Associated Network Hyperexcitability (LEV-AD) trial, failed to meet its primary and secondary endpoints; the former was a test of executive function and the latter were tests of cognition and function. However, in a prespecified exploratory analysis of participants with seizures or SEA, they found that treatment improved two of seven measures tested—a test of executive function (among 9 participants tested) and one of spatial memory (5 participants) (9). Moreover, recent findings indicate that neuronal hyperexcitability in patients with AD may be initiated by suppression of glutamate reuptake, which may suggest a novel therapeutic pathway for SEA in AD (33).

### High frequency oscillations

In addition to SEA and seizures, high frequency oscillations (HFOs) may also be seen on EEG in AD. Most EEG activity falls below a frequency of 30 Hz, but “fast ripples” between 250 and 500 Hz generally only occur at the time of seizure onset in patients with epilepsy—these are termed HFOs (34). In a recent study exploring these phenomena in AD, HFOs were detected in the hippocampi of all 3 AD mouse models evaluated but not in age-matched controls. Although human studies are needed, this novel EEG abnormality in AD may serve as a spatial biomarker for epileptogenicity in patients with AD and may suggest risk of AD development in patients with epilepsy (35).

## Seizure semiology and clinical course in Alzheimer’s disease

The predominant seizure type in patients with AD is focal non-motor onset seizures with impaired awareness (29, 36). These seizures may be characterized by an aura (e.g., déjà vu, unexplained emotions, and/or sensory phenomena), impairment in consciousness, and other common seizure semiology (e.g., staring, speech arrest, or memory loss). Patients with AD can also have generalized tonic–clonic seizures, as well. In 10 early onset Alzheimer’s disease (EOAD) patients with epilepsy, seizure types included generalized onset tonic-clonic seizures (25%), temporal lobe seizures (25%), myoclonus (25%), focal onset extra-temporal seizures (8%), and other types (17%) (37).

In a study that evaluated National Alzheimer’s Coordinating Center (NACC) data to determine the clinical course of seizures in patients with AD, the authors identified a 70% seizure recurrence rate within an average of 8 months of follow-up. Patients with AD and seizures had an earlier onset of cognitive impairment (mean age 65) compared to patients with AD who did not have concomitant seizures (mean age 70). The risk of seizures among patients with AD increased by an average of 0.64% per year (38). A comprehensive review of medical records that identified 1,320 patients with concomitant AD and unprovoked seizures similarly identified an increased seizure risk in patients with an early age of onset of AD and identified this relationship with myoclonus as well. Additionally, the probability of myoclonus increased gradually over time in individuals with AD. Seizures and myoclonus often co-occurred, and the authors suggest that the presence of myoclonus can guide earlier detection of seizures (39).

## Cognitive impairment due to epilepsy

Cognitive impairment associated with epilepsy can occur independently of AD. This has implications for providers monitoring for cognitive decline in patients with AD who develop epilepsy and in patients with AD where cognitive decline is progressing more steeply than anticipated. Studies show that cognitive scores for 257 participants aged 12–62 years with epilepsy for an average of 7 years were lower for the Montreal Cognitive Assessment (MoCA) and the Clinical Memory Scale compared to healthy controls (40, 41). In a study examining the association between late-onset epilepsy (LOE) and changes in cognitive performance over 25 years, 585 participants (average age 59.4 years) with LOE showed significant cognitive decline in global cognition, verbal memory, executive function, and word fluency compared with healthy non-LOE participants (42). Unsurprisingly, adult patients with temporal lobe epilepsy have more impaired episodic memory compared with those with other regional epilepsy syndromes (43). On the other hand, frontal lobe epilepsy often affects executive function and working memory long-term; additionally, in a systematic review of 35 studies, there was an association between cognitive changes and psychiatric symptoms in nearly 35% of participants with frontal lobe epilepsy (44, 45).

## Use of antiseizure medications in patients with comorbid Alzheimer’s disease and epilepsy

Lamotrigine and levetiracetam are both in common use for the treatment of seizures in patients with Alzheimer’s disease (46–48). Given promising results in preclinical rodent models, levetiracetam has also been evaluated as a treatment for cognitive impairment in participants with AD in the LEV-AD study detailed above. The study included participants with and without seizures and/or epileptiform discharges. As mentioned, there was no difference between treatment and placebo groups on the primary or secondary outcome measures of cognition and function. Treatment did appear to improve executive function and spatial memory, however, in an exploratory subgroup analysis of those with epileptiform discharges or seizures (9).

Of note, a randomized, double-blind, placebo-controlled study of 16 healthy participants aged 18–50 comparing acute dosing of brivaracetam 10 mg, levetiracetam 500 mg, lorazepam 2 mg, and placebo determined that lorazepam adversely affected the cognitive neurophysiologic test (CNT) score, which is a combination of EEG monitoring, evoked potential recordings, and cognitive performance measures. Brivaracetam did not differ from placebo or levetiracetam on any cognitive measures (10). An important association has been identified in Finnish and German registries between regular use of certain antiseizure medications (ASMs), namely phenobarbital, carbamazepine, phenytoin, valproate, primidone, barbexaclone, ethosuximide, clonazepam, zonisamide, and topiramate, and a greater risk of incident all-cause dementia (28%) and AD (15%). This analysis was adjusted for multiple confounders including a history of epilepsy. When the above medications were compared with a separate set of ASMs, including levetiracetam, oxcarbazepine, lamotrigine, gabapentin, vigabatrin, pregabalin, tiagabine, and lacosamide, the risk of dementia and AD was higher in the former group (11). Additional studies are needed to determine whether a causal inference can be made. In a study of 95 patients with AD and epilepsy, levetiracetam had fewer adverse effects than lamotrigine or phenobarbital. Of note, levetiracetam surprisingly increased MMSE scores (albeit by a modest 0.23 points), and lamotrigine improved mood (12). However, results have varied. A recent retrospective study compared 19 patients with epilepsy and mild cognitive impairment due to AD to 16 patients with MCI due to AD who did not have epilepsy. Nearly 90% of the patients with epilepsy were well-controlled with monotherapy, with seizure control defined as >50% seizure reduction. However, patients required an average of 2 lines of therapy due to adverse events or lack of seizure control. The top two main ASMs based on tolerability and efficacy were lamotrigine in 9 patients and lacosamide in 3 patients. In contrast to the above referenced study, levetiracetam was discontinued in 5 of 5 patients in this group due to adverse events including mood changes, mental slowing, asthenia, apathy, and aggressiveness (49).

## Effect of commonly used treatments in Alzheimer’s disease on seizure threshold

### Antidepressants

The prevalence of depression in AD approaches 15%, and antidepressants are commonly prescribed (50). The use of antidepressant drugs may increase risk of epilepsy, and there are unique treatment considerations in patients with co-morbid AD and epilepsy. In a study of 238,963 patients with a diagnosis of depression (age 20–64) taking antidepressants, the hazard ratio for seizures for all antidepressant drug classes significantly increased. Trazodone, lofepramine, and venlafaxine carried the highest risk compared to no treatment (13). A case-control study of 151,005 patients with depression who were prescribed selective serotonin reuptake inhibitors (SSRIs) or selective norepinephrine reuptake inhibitors (SNRIs) were two times more likely to be diagnosed with first-time seizures compared with non-users. Use of low-dose tricyclic antidepressants was not associated with seizures (14). However, tricyclics are often avoided in patients with AD due to their anticholinergic properties. Longer treatment duration with antidepressants is also associated with higher epilepsy risk (14). Thus, in patients with AD, epilepsy, and depression, an SSRI may be the safest pharmacologic option for treatment of depression with careful reassessment of the need for ongoing treatment at regular intervals.

### Antipsychotics

Although there is an FDA black box warning for the use of antipsychotics in patients with AD dementia, they are sometimes necessary when verbal and physical agitation or delusions cannot be redirected, do not respond to alternate therapies, and present a safety concern. Patients with dementia using olanzapine, quetiapine, low-to-medium potency first-generation antipsychotics, and medium-to-high potency first-generation antipsychotics have a higher risk of seizures compared to their counterparts who are not on these medications. In contrast, the use of amisulpride, aripiprazole, risperidone, or sulpiride does not have an association with increased seizure risk (15). In patients who co-morbid AD and epilepsy who require a medication in the antipsychotic class, these latter options may be optimal; however, the presence of parkinsonism may nonetheless prompt consideration of medications like quetiapine to lower the risk of extrapyramidal side effects.

### Acetylcholinesterase inhibitors

Acetycholinesterase inhibitors, including donepezil, rivastigmine, and galantamine, are commonly prescribed for the treatment of cognitive impairment in mild to severe dementia due to AD. There is a loss of cholinergic innervation from the basal forebrain to the cortex in patients with AD, and this forms the basis of the “cholinergic hypothesis” that some of the cognitive and behavioral symptoms in patients with AD are due to loss of cholinergic inputs that can be ameliorated with acetylcholinesterase inhibitors (51, 52). In a randomized, double-blind, placebo-controlled trial of donepezil to improve memory in epilepsy, 23 patients with epilepsy (ages 18–55) with subjective cognitive impairment were randomized to 3 months of donepezil (10 mg/day) or 3 months of placebo treatment. Each arm then crossed over to the other treatment group. The inclusion criteria included patients with definite epilepsy, the use of ASMs for epilepsy, and reports of memory concerns at the time of enrollment. Donepezil treatment did not result in a change in seizure frequency or severity. Additionally, treatment did not result in any significant changes in memory scores or other cognitive scores (16). In a separate population-based study, 13,767 participants aged 65–95 years who experienced incident seizures with dementia (Alzheimer’s dementia or vascular dementia) and prescribed donepezil, rivastigmine, galantamine, or memantine, there was a slight increase in seizure risk for patients receiving donepezil for 1 year compared to memantine. The mechanism is unclear, but off-target reductions in cortical dopamine and serotonin have been proposed (17).

### N-methyl-D-aspartate receptor antagonists

Memantine, an N-methyl-D-aspartate (NMDA) receptor antagonist commonly used in the treatment of moderate to severe dementia due to AD, has previously been shown to reduce seizure severity and duration at certain doses in rodents while inducing seizures in rats with kindled amygdalae at higher doses. As mentioned above, memantine seems to have a better profile in terms of seizure risk compared with donepezil (17).

### Anti-amyloid antibodies

Aducanumab and lecanemab are anti-amyloid antibodies that have both been granted FDA approval for the treatment of Alzheimer’s disease; the former was granted accelerated approval, and the latter was granted full approval. In the phase 3 clinical trials EMERGE and ENGAGE for aducanumab, 10.6% of patients who received the high 10 mg/kg dose had recurrent amyloid-related imaging abnormalities (ARIA) most of whom were asymptomatic. Seizures attributed to ARIA were reported in 0.4% of patients treated with the high dose. The overall incidence of seizures was balanced between the aducanumab and placebo groups (53, 54). In a phase 2 study of lecanemab, there was a single case of ARIA with edema (ARIA-E) associated with seizure (55). The phase 3 CLARITY AD trial for lecanemab did not mention seizures (56). Appropriate use recommendations for lecanemab mention that this drug should not be prescribed in patients with seizures since Clarity AD excluded patients who had seizures within the 12 months prior to screening (57). Another anti-amyloid antibody, donanemab, has demonstrated clinical efficacy in AD but is not yet FDA approved. Like aducanemab and lecanemab, donanemab carries the risk of ARIA, which can lead to seizures (58).

### Anti-tau antibodies

Since epileptogenesis in AD is hypothesized to be at least partly tau-mediated, anti-tau monoclonal antibodies hold theoretical promise as a treatment for co-morbid AD and epilepsy. Currently, there are no FDA approved anti-tau antibodies for AD. To date, monoclonal antibodies targeting tau in individuals with AD, including semorinemab, gosuranemab, tilavonemab, and zagotenemab, have failed in major clinical trials (59). In a mouse model of genetic tau reduction in aged mice, tau reduction increased resistance to seizure (60). This work suggests that treatments targeting tau present a critical future direction for research focusing on treatments that dampen hyperexcitability in AD.

## Active clinical studies and future directions

Ongoing trials aim to further explore the effects of levetiracetam on seizures and abnormal discharges in AD. Levetiracetam for Alzheimer’s Disease Neuropsychiatric Symptoms Related to Epilepsy Trial (LAPSE) is a phase 2 study wherein investigators intend to recruit 65 participants with probable AD and epileptiform activity identified on EEG and exclude participants previously diagnosed with epilepsy. Participants will take 500 mg twice a day for 1 year and will complete up to 3 serial EEGs. The primary outcome measure for this study is change in the Neuropsychiatric Inventory Score, or NPI (NCT04004702). Another clinical trial in the pipeline is an Investigation of Levetiracetam in Alzheimer’s Disease (ILiAD), and the primary outcome is a computerized hippocampus-dependent memory-binding test. This study will randomize 30 participants 50 years and older diagnosed with mild to moderate AD and will exclude participants with a diagnosis of epilepsy. The treatment arm consists of uptitrating levetiracetam by 250 mg at one-week intervals to 1 g twice daily for 4 weeks followed by downtitration over 4 weeks (NCT03489044).

There are a few additional proposed prospective studies that aim to characterize patients with both Alzheimer’s disease and epilepsy. The Prevalence of Epilepsy and Sleep Wake Disorders in Alzheimer Disease (PESAD) study aims to perform 48-h ambulatory scalp EEGs and polysomnograms in 100 individuals with AD and 30 gender- and age-matched healthy individuals for early detection of epilepsy and sleep–wake disturbances. Fifteen participants with AD who have epileptic spikes or sleep–wake disorders will undergo invasive EEG monitoring to evaluate for the presence of hippocampal seizures. Current and future findings may support whether early development of hippocampal hyperexcitability is a precursor to cognitive decline in AD (NCT03617497).

Since there is overlap in AD and epilepsy pathogenesis, studies are also examining the predictive value of biomarkers. One trial examining the profile of CSF biomarkers in AD, which is called the Predictive Value of Biomarkers of Alzheimer’s Disease in Elderly Patients with New-onset Epilepsy (BIOMALEPSIE) study, aims to recruit 35 cognitively normal patients older than 60 years with new-onset epilepsy. Investigators hypothesize that elderly participants with new epilepsy diagnoses will have more amyloid pathology than their healthy counterparts (NCT02861846). A final upcoming study aims to explore the prevalence of SEA in the hippocampus in patients with CSF-proven MCI due to AD compared to healthy controls and track its role in clinical progression over 2 years (EADP study, NCT04131491).

## Discussion

Patients diagnosed with AD have a higher risk of seizures compared with counterparts without AD, and patients diagnosed with epilepsy are at increased risk of AD. This bi-directional relationship may be explained by the shared neuropathology of AD and epilepsy, including a decrease in Aβ42 in CSF and an increase in hyperphosphorylated tau protein (19, 21, 22). There is also limited evidence for hippocampal hyperactivity in AD, which may negatively affect memory consolidation (31, 61). Epilepsy is associated with cognitive impairment on its own, and the dual diagnoses of AD and epilepsy may compound the cognitive decline characteristic of AD. Researchers have considered whether cognitive impairment in AD could be treated with ASMs, but a small study of levetiracetam in participants did not result in a cognitive benefit unless participants had epileptiform activity (9). Nonetheless, that study may have been limited by its small size, difficulties with recruitment, and heterogenous patient population in terms of the presence or absence of epileptiform activity. Given these limitations, the door remains open for an antiseizure drug to provide meaningful clinical benefit in patients with AD, and there are currently two active trials examining the effects of levetiracetam on neuropsychiatric features and cognitive impairment in AD via the LAPSE and ILiAD trials (NCT04004702, NCT03489044).

Several medications that are frequently prescribed for patients with Alzheimer’s disease can affect seizure threshold, and thus, there are unique treatment considerations in patients with co-morbid epilepsy and AD. Among antidepressants, trazodone, lofepramine, and venlafaxine may be most likely to lower seizure threshold. Among neuroleptics, olanzapine, quetiapine, and first-generation antipsychotic drugs are most likely to increase seizure risk and may need to be avoided in patients with both AD and epilepsy. Also, there is some limited evidence that the rapid withdrawal of acetylcholinesterase inhibitors may lower seizure threshold. Understanding the impact of these commonly used medications on seizure risk can guide clinical decisions for patients with co-morbid AD and epilepsy. Anti-amyloid monoclonal antibodies are not recommended for use in patients with seizure activity, particularly in patients with seizures within the year prior to initiation of therapy, given the risk of seizures associated with ARIA in drugs in this class. However, the study of anti-amyloid antibodies in combination with ASMs represents a potential, as yet unexplored direction for AD with co-morbid epilepsy. Anti-tau antibodies also hold theoretical promise in this patient population.

The ongoing PESAD study seeks to identify the prevalence of epileptiform discharges and seizures in AD via prolonged EEG monitoring, the BIOMALEPSIE trial aims to understand the AD biomarker profile of patients with late-onset epilepsy (NCT03617497, NCT04131491), and the EADP study will characterize SEA in patients with MCI due to AD. These studies are essential given that a better understanding of the underlying shared mechanisms of AD and epilepsy can be used to guide the development of novel therapies in the clinical pipeline.

## Author contributions

OL, TK, and IS-S equally contributed to the drafting and final version of the entire manuscript. All authors contributed to the article and approved the submitted version.
